# The Medicinal Value of the Yellow Marigold

**Published:** 1891-08

**Authors:** 


### THE MEDICINAL VALUE OF THE YELLOW MARIGOLD.

Doubtless few of our readers have any conception of the great medicinal value of the flower of the common yellow marigold, known in medicine as *calendula*, for cuts, superficial wounds, and bruises ; we know of nothing as an external application, nearly so meritorious as this simple garden flower. It is far superior to arnica, although the latter possesses great merit. We advise every reader in whose garden the yellow marigold may grow to carefully pick this flower and dry a good supply of the flowers each season. These flowers, when thoroughly dried, should be placed in a bottle and covered with diluted alcohol—that is, one-half alcohol and one-half water ; keep well corked. When needed for cuts, wounds, or bruises, put a tea-spoonful of this tincture in a half cup of water, and saturate a cloth in this solution and bind round the wounded part : it may smart slightly for a moment, but this soon passes away, when in a short time the injured part will be cured.

